# Spontaneous Intravesical Knotting of Urethral Catheter

**Published:** 2011-11-27

**Authors:** Yogesh Kumar Sarin

**Affiliations:** Department of Pediatric Surgery, Maulana Azad Medical College, New Delhi-110002

**Keywords:** Urinary catheter, Catheterization complication, Intravesical knotting, Posterior sagittal anorectoplasty

## Abstract

Infant feeding tubes (IFT) have been universally used as urethral catheters in neonates and children for several decades. Though generally a safe procedure, it may cause significant morbidity if the catheter spontaneously knots inside the bladder. We report this complication in three children including a neonate.

## INTRODUCTION

Catheters inserted for various purposes, urological as well as non-urological, are known to rarely knot spontaneously inside the human body with an estimated incidence of 0.2 per 100,000 catheterizations [1]. Raveenthiran could find only 40 cases of knotted urinary catheters on a recent review of the world literature [2]. The report intends to generate awareness of a potentially preventable complication that can result in significant morbidity with a list of recommendations to minimize this risk.

## CASE REPORT

**Case 1:** An eight-month-old male infant, a case of anorectal agenesis with rectoprostatic urethral fistula with status sigmoid loop colostomy, underwent posterior sagittal anorectoplasty. He was catheterized with a 6 Fr infant feeding tube intra-operatively. The surgery and the post-operative period were uneventful. Gentle traction on the catheter however failed to retrieve the catheter on seventh post-operative day. On examining along the urethra, the knotted catheter could be palpated at the perineum. Pelvic roentgenogram confirmed the diagnosis of knotted catheter in the urethra. Several attempts at forceful introduction of sterile saline and contrast material under fluoroscopy failed to unwind the loop.

Under short dissociate anesthesia, another attempt was made to untie the knot and straighten the catheter with angiography wire through the catheter lumen. Failing this maneuver, the catheter was divided flush with the glans penis and the knotted catheter was gently manipulated out through a small perineal urethrostomy (Fig. 1). A percutaneous suprapubic tube was inserted and was left in place for a week. The child had been passing urine in good stream on follow up.

**Figure:**
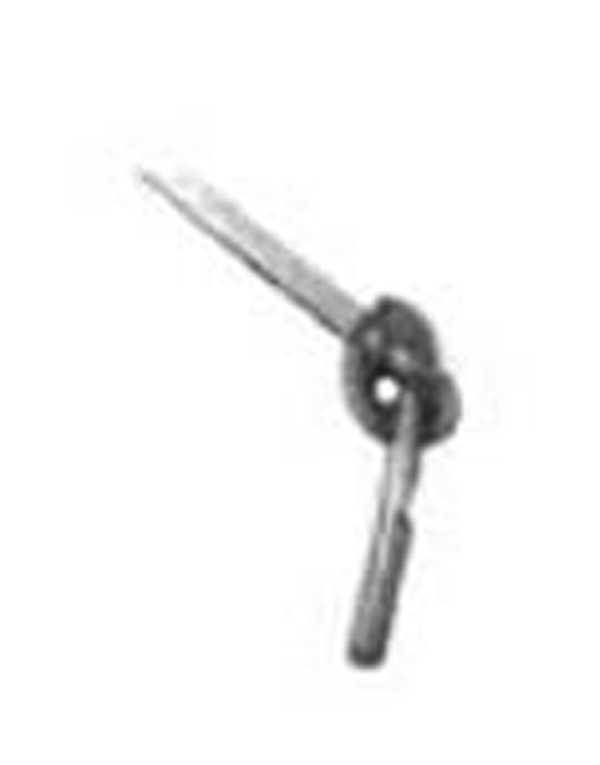
Figure 1: Knotted catheter after retrieval through perineal urethrostomy

**Case 2:** A male newborn weighing 1.8 kg was operated for ileal atresia on day-1 of life; resection of atretic segment and end-to-back ileo-ascending colic anastomosis was done. The patient was re-operated after two weeks for anastomotic leak. Three days later, an attempt to remove the catheter (6 Fr IFT) was met with resistance. From the past clinical experience, catheter knotting was suspected. On this occasion, manipulation with angiography wire through the catheter lumen succeeded and the catheter removed. The child was discharged after a month of admission. Unfortunately, a week later he was brought moribund to the casualty where he succumbed to severe dehydration and refractory shock.

**Case 3:** One and half year old boy underwent endoscopic valve incision for posterior urethra valves. The child was lost to follow-up for 5 years when he presented again with poor urinary stream. He could not be catheterized and was diagnosed to have urethral stricture at bulbo-membranous junction on retrograde urethrography. Endoscopic incision of hypertrophied bladder neck and visual internal urethrotomy of stricture was done; there were no residual posterior urethral valves. Three days later, an attempt to remove the catheter was met with resistance. The catheter was removed using local and systemic analgesia and gentle steady traction. The tip of the catheter was found knotted. The patient voided clear urine spontaneously and comfortably after few hours. He later underwent endoscopic management of bilateral major grades of vesico-ureteral reflux (deflux injection). He is under close follow up.

## DISCUSSION

Intravesical knotting of catheters have been reported more commonly in males than females, and more commonly in neonates and children than adults [2]. Intravesical knotting has been reported not only in catheters left for bladder drainage, but also after brief maneuvers such as clean intermittent catheterization, and cystourethrography [3, 4, 5]. This is the first instance that this complication has been encountered following posterior sagittal anorectoplasty.

Although knotting of urethral catheters is rare, removal represents significant morbidity, such as general anesthesia, radiation exposure during fluoroscopy, and transient hematuria [1]. Potential for further complications such as stricture formation also needs to be considered. Knotted urinary catheters may also jeopardize delicate surgical reconstructions [3, 6]. Unfortunately, many doctor colleagues and nursing staff are unaware of this problem or its proper management. A telephone survey of 24 tertiary- care Emergency Departments in Canada revealed that none of them were aware of catheter knotting and 22 had no protocol established for safe catheterization [1].

Several hypothetical explanations have been offered for the knotting of catheters. The tendency of a catheter to knot probably depends on its flexibility, smaller diameter and redundancy within the bladder. The probable mechanism involves an extra length of catheter coiling around itself and then the catheter end looping through these coils [4]. The coils tighten cinching down in a knot when counter traction is applied to remove the catheter. If the diameter of this knot exceeds that of urethra the catheter gets stuck. Bladder spasm has been also been attributed as a risk factor [7]. Water-current generated by the flow of urine around the catheter may also play a role in the genesis of catheter knotting [2]. Raveenthiran suggested that the catheters slender than 10 Fr, over-distended bladder and insertion of excessive length (greater than 10 cm beyond bladder neck) of catheters must be considered as risk factors for catheter knotting [2].

Several techniques have been described to retrieve the knotted catheter. They include sustained traction under anesthesia, unraveling the knot using a guide-wire through the catheter under fluoroscopy, endoscopic retrieval and suprapubic cystotomy [4, 7, 8, 9]. Guide-wire manipulation is useful only at the early ‘open-loop stage’ of knot formation when the knot is not tight enough [8] and succeeded in one of our cases. Sustained traction also worked once, but such a manipulation with or without urethral dilatation carries the risk of urethral damage. Moreover, this technique is not useful when the knot is bulky or when two catheters knot together [2]. Suprapubic cystotomy has been known as a simple, safe and cost-effective method of retrieving knotted bladder catheters [10], though in the modern era, this could be replaced by vesicoscopy.

The attention should be directed towards prevention of this complication by careful selection of the catheters and gaining better understanding of urethral anatomy and safe insertion lengths. The insertion lengths of 6 cm in a male newborn and 5 cm in a female newborn have been recommended [10]. In extremely premature babies with birth weight of &<750 grams the insertion length of &<2.5 cm in girls and &<5 cm in boys is recommended [10]. It is also equally important to secure the catheter well in order to prevent inadvertent advancement of the catheter into the bladder [1]. 

## Footnotes

**Source of Support:** Nil

**Conflict of Interest:** None declared
